# C22 podovirus infectivity is associated with intermediate stiffness

**DOI:** 10.1038/s41598-020-69409-w

**Published:** 2020-07-28

**Authors:** Udom Sae-Ueng, Anjana Bhunchoth, Namthip Phironrit, Alongkot Treetong, Chaweewan Sapcharoenkun, Orawan Chatchawankanphanich, Ubolsree Leartsakulpanich, Penchit Chitnumsub

**Affiliations:** 1 National Center for Genetic Engineering and Biotechnology (BIOTEC), National Science and Technology Development Agency (NSTDA), 113 Thailand Science Park, Phahonyothin Road, Khlong Nueng, Khlong Luang, Pathum Thani, 12120 Thailand; 2 National Nanotechnology Center (NANOTEC), National Science and Technology Development Agency (NSTDA), 111 Thailand Science Park, Phahonyothin Road, Khlong Nueng, Khlong Luang, Pathum Thani, 12120 Thailand

**Keywords:** Nanoscale biophysics, Applications of AFM

## Abstract

Bacteriophages have potential for use as biological control agents (biocontrols) of pathogenic bacteria, but their low stability is limiting for their utilization as biocontrols. Understanding of the conditions conducive to storage of phages in which infectivity is maintained over long periods will be useful for their application as biocontrols. We employed a nanomechanical approach to study how external environmental factors affect surface properties and infectivity of the podovirus C22 phage, a candidate for biocontrol of *Ralstonia solanacearum,* the agent of bacterial wilt in crops. We performed atomic force microscopy (AFM)-based nano-indentation on the C22 phage in buffers with varying pH and ionic strength. The infectivity data from plaque assay in the same conditions revealed that an intermediate range of stiffness was associated with phage titer that remained consistently high, even after prolonged storage up to 182 days. The data are consistent with the model that C22 phage must adopt a metastable state for maximal infectivity, and external factors that alter the stiffness of the phage capsid lead to perturbation of this infective state.

## Introduction

*Ralstonia solanacearum*, the agent of bacterial wilt disease in crops such as potato, tomato, tobacco and chili, is ranked as the second most important bacterial pathogen worldwide, responsible for an estimated annual loss of crop value exceeding 950 million US dollars^1,2^. Chemical methods for controlling *R. solanacearum* are ineffective and environmentally damaging. Natural biological control agents, also known as biocontrols, represent an alternative pest control method that is sustainable, more effective and less detrimental to the environment. Bacteriophage viruses (phages) that naturally infect bacterial pathogens in soil are attractive biocontrol candidates. Phage biocontrol is sustainable because it does not introduce foreign materials into the environment^3^. Secondly, phages can only replicate in specific host bacteria, meaning that once all host cells are lysed, phage replication will terminate^4,5^. Thirdly, phages are non-toxic to humans, rendering them appropriate for consumable products^6^. Despite the advantages of phages for biocontrol, phages must remain viable and infectious for long periods, including storage and field application^7–9^. The stability of phage is therefore a critical issue that compromises its practical use in agriculture. The optimal conditions to maintain phage infectivity are thus important for efficient phage usage in biocontrol.

Phage survival and infectivity are dependent on the nanomechanical property of the phage genomic material encapsulated by the capsid protein shell^10,11^. It has been shown that DNA packaging^12^, DNA ejection from the capsid^13,14^, and DNA transition inside the capsid^15^ are correlated with phage nanomechanical property. We recently isolated a novel phage named C22 in the family of *Podoviridae* (podovirus) that can lyse *R. solanacearum*. The high lytic activity of this phage makes it an attractive candidate for biological control of *R. solanacearum*^16^. In this study, we characterized the nanomechanical property of C22 phage by measuring stiffness using atomic force microscopy (AFM). Employing a nanometer-size tip, AFM can acquire stiffness data and nanometer-resolution images of phage in a liquid milieu^17–20^. Therefore, the data reflect the phage’s properties in its natural infectious state. We found that C22 phage exhibited variable stiffness according to the conditions of buffer pH and ionic concentration, and that phage stiffness was associated with phage infectivity. The data suggest that intermediate stiffness, possibly representing a metastable state, is key for phage stability.

## Results

### Nanomechanical property of C22 phage

**Figure 1:**
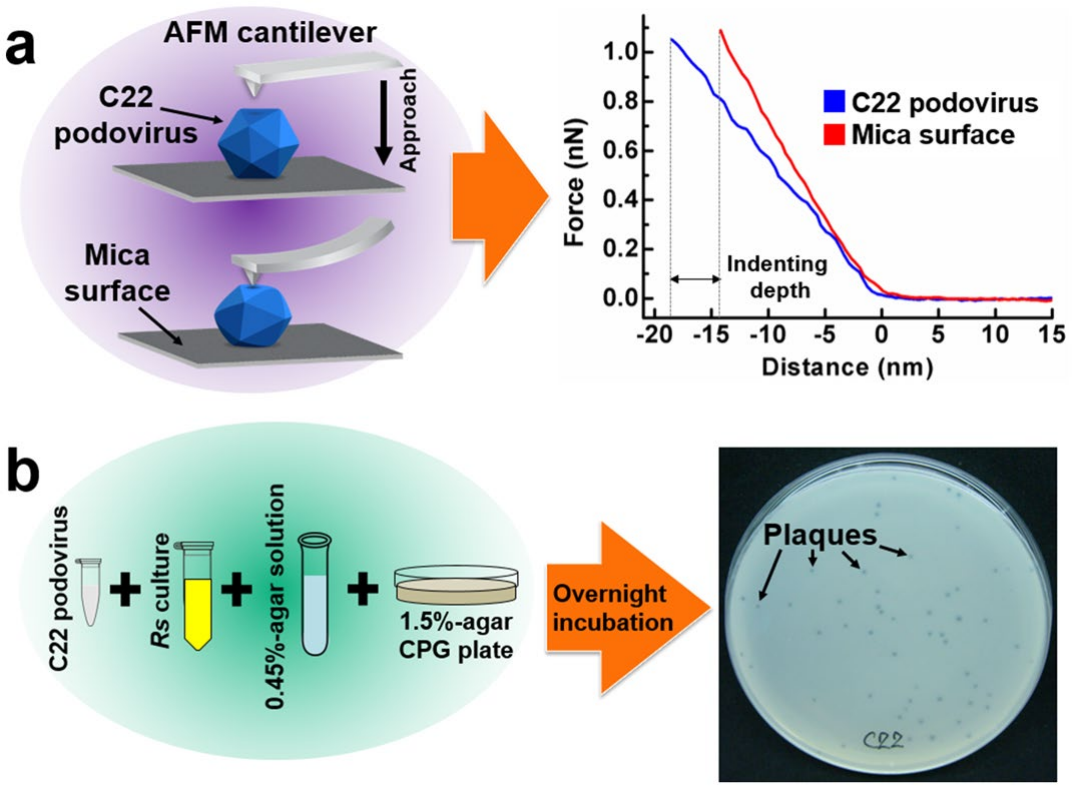
Experimental schemes of AFM-based nano-indentation and plaque assay. (**a**) C22 virus particles (blue) were bound to a chemically treated mica surface (dark grey). An AFM cantilever (light grey) approaches the particle by moving downward and indents into the particle. The indentation on the particle (blue) and on a mica surface (red) yields force-distance curves for the stiffness calculation of the C22 phage. The separation between the two force-distance curves determines the indenting depth into the phage particle. (**b**) Plaque assay performed by the double agar layer technique. 1.5% agar CPG was overlaid with a top layer of 0.45% agar CPG containing *Ralstonia solanacearum* (*Rs*) and C22 phage. After overnight incubation, clear zones (plaques) from the bacterial lysis were observed and enumerated (black arrows).

**Figure 2:**
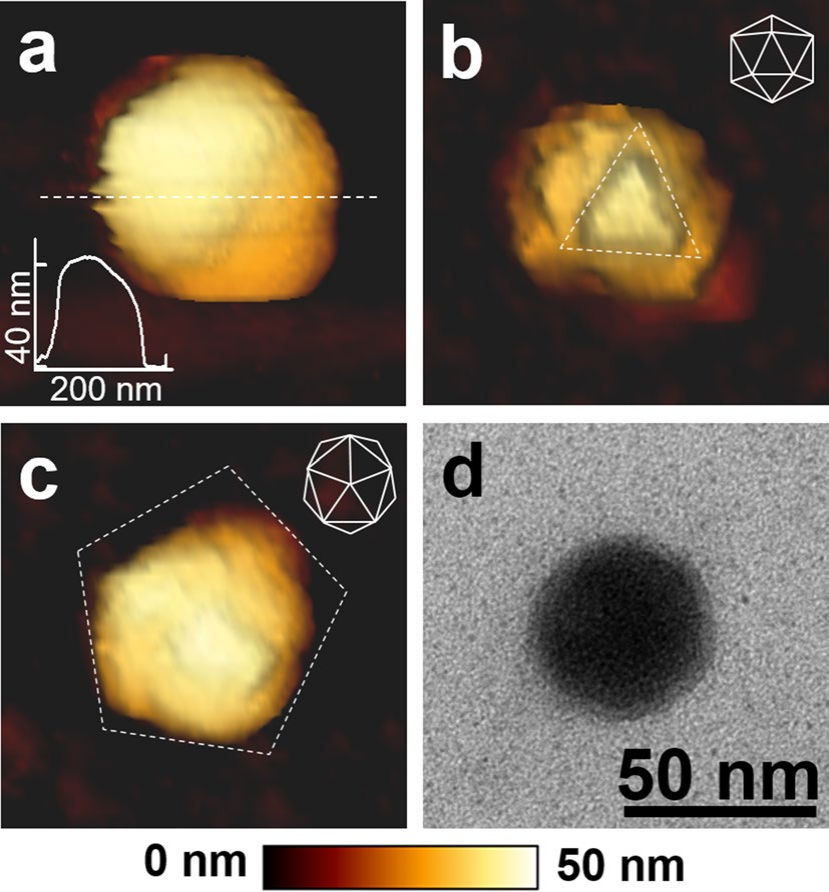
Microscopic images of the C22 phage. AFM images are shown in parts (**a**–**c**). (**a**) Analysis of the cross section indicates the C22 phage diameter to be 40 nm. (**b**) and (**c**) Images showing the threefold and fivefold symmetry faces, respectively consistent with icosahedral geometry of the phage capsid (schematic drawing top right). The color scale bar underneath the images is the scale in the vertical (Z) axis for the AFM images. (**d**) TEM image of a C22 phage particle; scale bar = 50 nm.

**Figure 3:**
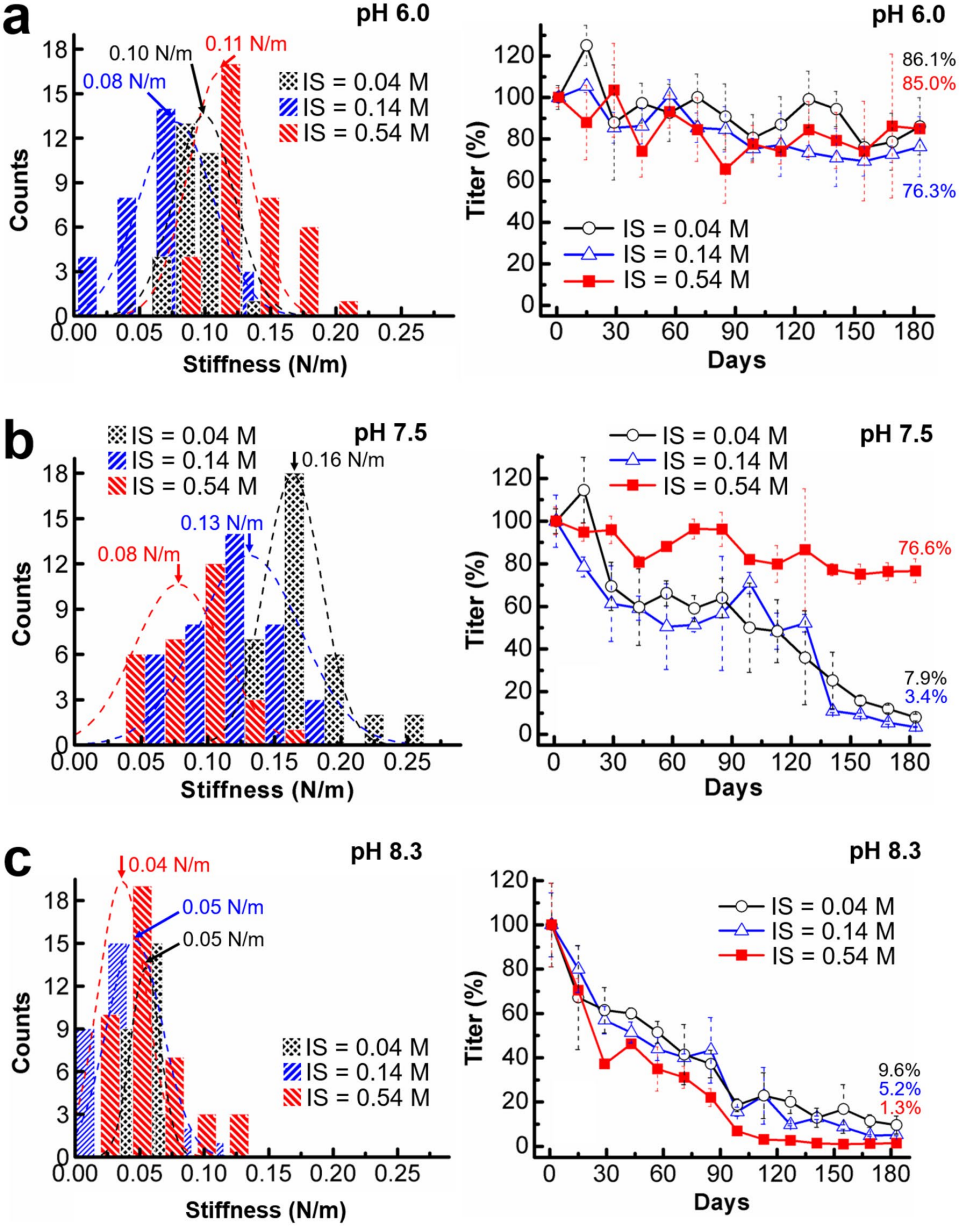
Plots of the stiffness distributions (left) and the titers (right) of the C22 podovirus in the buffers at pH 6.0 (**a**), 7.5 (**b**), and 8.3 (**c**) with the ionic strengths (IS) of 0.04 M, 0.14 M, and 0.54 M. (Left) The dashed lines are the Gaussian fitted distributions where the peak centers represent the phage stiffness. (Right) Phage titers were monitored in triplicate at 14 day intervals. Error bars represent standard deviations (s.d.).

The nanomechanical property of phage can be measured by AFM-based nano-indentation, in which the phage properties are probed via the direct interaction between the AFM tip and the surface of the phage structure as shown in Fig. 1a. C22 phage images were captured using AFM in the non-contact imaging mode (Fig. 2a–c). C22 particles exhibited icosahedral geometry with a diameter of 40 nm (Fig. 2a inset) consistent with the transmission electron microscope (TEM) image (Fig. 2d). The threefold and fivefold symmetry faces observed in AFM images (Fig. 2b,c) were consistent with the characteristic features of viral icosahedral geometry^21,22^. The AFM images of C22 particles (Fig. 2a–c) appeared to map incompletely to the icosahedral outline (insets), which is likely due to occasional aberrant tip-sample interactions of soft biological samples in the non-contact imaging mode^23,24^. We then explored the nanomechanical property of the C22 phage in SM buffer, a buffer generally used to store phage^16,25,26^. Using the springs-in-series model^18,27,28^ and Gaussian distribution fitting, the mean stiffness of the C22 phage in SM buffer was revealed to be 0.13 N/m. With this technique, we measured the stiffness of C22 phage particles in buffers with varying pH and ionic strength to understand the phage nanomechanical responses under different conditions (Fig. 3, left column and Supplementary Table 1).

Previous nanomechanical studies on viruses have shown that the mechanical properties of viral capsids, e.g. stiffness depend on the capsid orientation of symmetry faces^29,30^. We tested the stiffness dependence on the capsid orientation of the C22 phage. The AFM images of the C22 phage capsids displayed three orientations—twofold, threefold, and fivefold symmetry faces (Supplementary Fig. S1). The average stiffness of the C22 capsids in three buffers (pH 6.0, 7.5, and 8.3 with ionic strength of 0.14 M) representing three ranges of stiffness was recorded according to the symmetry faces (Supplementary Table 2). The stiffness of the C22 capsids with three symmetry faces in each buffer was essentially the same, indicating that the stiffness of the C22 capsids in our buffer conditions did not depend on the capsid orientation.

### Effect of pH and ionic strength on C22 phage stiffness and infectivity on *R. solanacearum*

The properties of the phage capsid protein and DNA vary according to environmental factors such as pH and ionic strength^31–33^. We tested the stiffness of C22 phage in various conditions of pH and ionic strength. We selected three buffers representing acidic (pH 6.0), neutral (pH 7.5) and basic (pH 8.3) conditions, and three different ionic strength conditions (0.04 M, 0.14 M, and 0.54 M) to cover the variation of pH and ionic strength in soils^34^, including the natural soil conditions of the C22 phage habitat (Supplementary Table 3).

In the acidic buffer (Fig. 3a, left), the mean stiffness values were 0.10 N/m, 0.08 N/m and 0.11 N/m at the ionic strengths of 0.04 M, 0.14 M, and 0.54 M, respectively. A larger variation of stiffness in neutral conditions was observed ranging from 0.08 to 0.16 N/m (Fig. 3b, left). The overlap of stiffness distributions under the acidic and neutral conditions suggests that the nanomechanical properties of C22 phage are mildly affected by pH within this range. In contrast, C22 phage stiffness was much reduced in alkaline conditions with mean stiffness ranging from 0.04 to 0.05 N/m among the ionic strengths tested (Fig. 3c, left).

To understand how buffer pH and ionic strength affect infectivity, we monitored the infectivity of the C22 phage on *R. solanacearum* by plaque assay for phage stored under different conditions for 182 days (Fig. 1b). Phage titer decreased to no less than 76.3% of the starting point at pH 6.0 (Fig. 3a, right). Under neutral conditions, phage titer remained high only at the ionic strength of 0.54 M, but decreased by more than 90% in conditions of lower ionic strength (Fig. 3b right). Phage titer decreased more than 90% under alkaline conditions at all ionic strengths tested (Fig. 3c, right). The results show that ionic strength and pH are critical for phage infectivity. The conditions for maximum C22 phage infectivity in phage stored for 182 days from this study were pH 6.0 with the ionic strength from 0.04 M to 0.54, and at pH 7.5 with the ionic strength of 0.54 M.

It is known that host cell condition, phage adsorption and phage aggregation are critical factors of phage infectivity that are affected by pH and ionic strength in addition to phage nanomechanical properties^9,35^. In this study, the *R. solanacearum* host condition was controlled by using the same cell cultures for plaque assays of C22 phage stored in all nine storage buffers. Moreover, the phage sample was overwhelmingly diluted by bacterial culture media in plaque assays such that the effect of phage storage buffer on host cell was negligible. We tested whether phage adsorption was affected by storage of C22 phage under different conditions of buffer pH and ionic strength by adsorption test to quantify the percent of the C22 phage attached to the host cell^35,36^. The adsorption test showed that the percentage of C22 phage attached to the host cell was not markedly different among the nine storage buffers (Supplementary Table 4). It is, however, important to note that C22 phage stored in each buffer condition can differently adsorb to the host cell at molecular level, but the difference may not be observed with our experimental setup. It is known that phage aggregation can be induced at a certain acidity or NaCl concentration^9,37^. However, no C22 phage aggregation was observed with AFM imaging, suggesting that aggregation is not the cause of the observed variation in C22 phage infectivity (Supplementary Fig. S2). While other factors induced by changing in pH and ionic strength may possibly influence or affect phage infectivity, the variation of the C22 phage stiffness suggests that the nanomechanical property and the infectivity of C22 phage particles are related.

**Figure 4:**
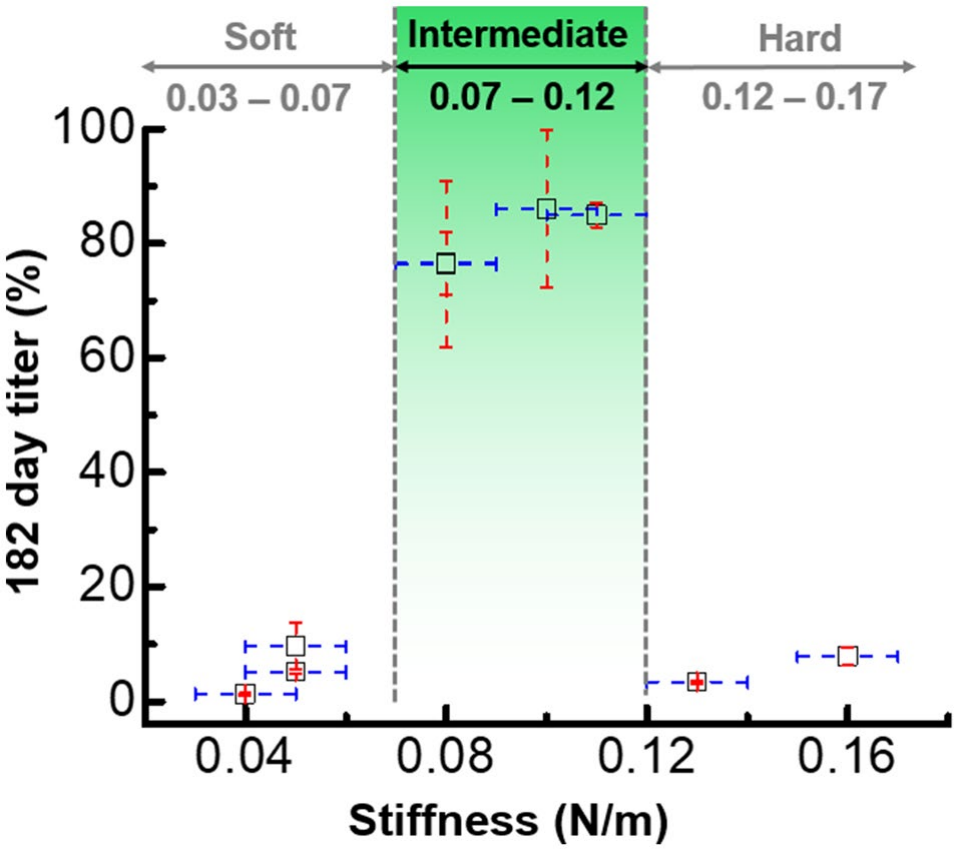
AFM data of C22 phage stiffness (x-axis) under different conditions of buffer pH and ionic strength combined with data of phage titer after storage for 182 day (y-axis). The stiffness values are extracted from Gaussian distributions (Fig. 3) containing 332 data points in total, and the titer values are calculated from 243 data points in total. The stiffness error bars represent the standard error, and the titer error bars represent the standard deviation.

To quantitatively validate the relationship between the stiffness and the infectivity, we performed Generalized Additive Models (GAM). GAM is a statistical model that is suitable for analysing unseen data sets since it allows nonlinearity in the data, proving to be a flexible model^38,39^. We collected 332 stiffness data points along with 243 values of titer. GAM was fitted on these data sets and showed a large value of R-Squared at 0.8837 with a very small p-value of less than 0.001 (Fig. S3). This indicates that 88.37% of the variation in infectivity can be explained by the stiffness with statistical significance of larger than 99%. For comparison, we also fitted GAM on the adsorption and infectivity data sets. The analysis result showed a low R-Squared of only 0.0425 with a small p-value (Fig. S4). With 95% significance, a very weak relationship between adsorption and infectivity is revealed. When the data of phage stiffness were combined with data of phage titer at 182 days, a pattern is evident in which high titer (> 80%) is found only in conditions that give rise to intermediate stiffness (0.07–0.12 N/m). In contrast, conditions that give rise to low (0.03–0.07 N/m) or high (0.12–0.17 N/m) stiffness are associated with low titer (< 10%) (Fig. 4).

## Discussion

In order to retain infectivity in changing natural environments, phages must structurally adapt to both physical and chemical changes by optimizing interactions among the capsomeres and encapsulated genome^40^. After elimination of host cell condition, phage adsorption and phage aggregation as factors responsible for changes in infectivity when C22 phage is stored in conditions of variable pH and ionic strength, stiffness was identified as a likely causal factor. The rapid decline of C22 phage titer at pH 8.3 (Fig. 3c, right), suggests that phage particles lose structural integrity owing to the reduction in stiffness in alkaline conditions (Fig. 3c, left). A similar observation was made for Norwalk virus, which loses capsid stability at pH 8; from this observation, it was proposed that non-covalent interactions of the capsomeres are influenced by pH^41^. The reduction of C22 phage titer and stiffness at pH 8.3 suggest a similar pH weakening effect of the capsomere interactions in C22 phage. In neutral conditions of low ionic strength, C22 phage exhibits high stiffness (Fig. 3b, left), potentially due to unfavorably tight capsomere interactions. This may induce a large stress on the phage structure and lead to loss of infectivity (Fig. 3b, right). In conditions that give rise to intermediate stiffness, C22 phage retains high titer and infectivity even after 182 days (Fig. 4), suggesting that the C22 proteinous capsid retains structural integrity in these conditions. The physico-chemical constraints on the interactions among capsomeres in proteinous capsid^22,42^ may be responsible for the narrow range of pH and ionic strength conditions compatible with C22 phage infectivity. Moreover, variations of pH and ionic strength can perturb charges around the double-stranded DNA (dsDNA) inside the phage capsid, which consequently alter external charge density of phage capsids and indirectly affect the mechanical properties of phage particles, as shown by previous AFM studies^43,44^.

Previous studies on mechanical stability of viral capsids have shown that the mechanical properties of phage capsids including stiffness, breaking force, and fragility are linked to chemical stability of phage capsids^45,46^. Therefore, the phage capsid of C22 phage with high stiffness may possess high chemical stability, including tolerance to changes in pH and ionic strength. However, chemical stability cannot explain the low infectivity of the C22 phage with high stiffness (0.12–0.17 N/m). Other puzzling roles of stiffness may be embedded in the mechanics-structure intrinsic relationship of viral capsids. The entanglement between nano-mechanics and structure has been hypothesized and validated in several viruses^47–49^. Here, it is possible that changing of the C22 phage stiffness is related structural alteration. Unfortunately, no apparent change such as disassembly or deformation in overall C22 capsid shape and diameter was observed in our pH and ionic strength conditions studied (Fig. S5 and Supplementary Table 5). The reason can also be the limitation of AFM in terms of high resolution imaging. Therefore, changing of the C22 phage morphology cannot be entirely excluded. Further structural study of C22 phage is needed to determine any capsid and capsomere modification triggered by changes in pH and ionic strength.

Previous studies on viral stiffness demonstrated that the mechanics-structure association is translated to relevance of viral infection^50^. The first work that showed prominent role of stiffness on viral infection process was conducted in Human Immunodeficiency Virus (HIV) where correlation between the softening of viruses during maturation and the efficiency of their host cell entry was revealed^51^. Recent studies on the minute virus of mice (MVM) unfolded that rearrangement of capsomeres into a so-called compact form of capsid with high stiffness led to substantially reduced infectivity^29,52^. Evidenced by the previous studies, we suggest that the stiffness of the C22 phage may play some direct and indirect roles in phage infection. The first possible role of the stiffness is to facilitate in adsorption at molecular level such as allowing better fitting between phage particle and host cell surface^53,54^. The second possible role of the stiffness is to promote of genome transfer into host cell^15,27^. Other possible unknown roles of stiffness remain to be explored. More studies are needed to unravel how viral infection mechanism may potentially be influenced by viral nano-mechanics, underlining the complexity, dynamics, and advanced designs of viral capsids.

It has been hypothesized that virus capsids try to “balance their stiffness to prevent inactivation without impairing infection”^55^. The intermediate stiffness of C22 phage revealed in this study agrees well with this hypothesis. The intermediate stiffness of C22 phage associated with maximal infectivity (Fig. 4) suggests that the capsid with this property has sufficient stiffness to preserve phage integrity, yet is sufficiently flexible to undergo conformational change as required during the infection process, consistent with viral metastability^56,57^. While the nano-mechanics of viral metastability are still little understood^58^, we propose the model that C22 phage with intermediate stiffness may therefore represent a metastable state in viral infection.

Metastability has been proposed to be an essential property of viruses in which they must be in a dynamical transient state that can protect them from external disruption while being able to effectively infect a host cell^40,56^. Previous studies on the metastability of the DNA state in lambda phage showed that the phage with high DNA stiffness at low temperature had a slow DNA ejection rate and potentially low infectivity, while low DNA stiffness at high temperature induces the transition of the DNA from the solid to fluid state during DNA release, potentially leading to high infectivity^15^. For enveloped viruses, the viral membrane has to be in a metastable state to withstand disruption from external physiological factors while flexible enough to interact with the host membrane or receptor^59,60^. Although C22 phage with intermediate stiffness is consistent with a metastable state, more data are needed to show the precise arrangement of capsomeres and how this arrangement is altered in different environments.

Our findings suggest that C22 phage stiffness can be used to predict phage infectivity and stability. This knowledge could be applied for testing conditions of phage storage and biocontrol, which will accelerate the development of podovirus and other phages for sustainable agriculture and therapeutic application.

## Methods

### Bacteriophage purification

An overnight culture of *Ralstonia solanacearum* strain RS3/1-1 (race 1 biovar 4) at 28 °C and 230 rpm shaking was diluted 100-fold with 200 mL fresh CPG medium containing 0.1% (w/v) casamino acids, 1% (w/v) peptone, and 0.5% (w/v) glucose supplemented with 10 mM MgSO_4_ in a 1-L flask^16^. When the cultures reached an OD_600_ of 0.3–0.4, C22 podovirus (isolated from a soil sample in a tomato crop field in Chiang Mai, Thailand) was added at multiplicity of infection of 0.05–0.1^16^. After an overnight incubation, clear culture was observed as a result of bacterial lysis. After adding 1 mg/mL DNase I (Roche) and 1 mg/mL RNase A (Sigma-Aldrich), the culture was incubated at 37 °C for 1 h. The cell debris was removed by centrifugation at 5,000 rpm for 30 min at 4 °C in a JA-14 rotor (Beckman Coulter). The supernatant was filtered through a 0.45 μm membrane (Corning) before being precipitated via centrifugation at 19,500 rpm for 40 min at 4 °C in a JA-30.50 Ti rotor (Beckman Coulter). The pellet was resuspended in SM buffer (50 mM Tris–HCl pH 7.5, 100 mM NaCl, 10 mM MgSO_4_, 0.01% (w/v) gelatin). The phage suspension was loaded onto a sucrose gradient (linear 50% to 20% sucrose in SM buffer) and subjected to ultracentrifugation at 24,000 rpm for 1 h at 4 °C in a TH-641 swinging bucket rotor (Thermo Fisher). The C22 podovirus appearing as a band between the 25%- and 30%-sucrose layers (Fig. S6) was extracted by puncturing on the side of the tube. The phage solution was tenfold diluted with the experimental buffer (Table 1) before being precipitated by ultracentrifugation at 24,000 rpm for 1 h at 4 °C in a TH-641 swinging bucket rotor (Thermo Fisher). The pellet was resuspended in the experimental buffer (Table 1) and stored at 4 °C. The characterization protocol with transmission electron microscope was described elsewhere^16^.

### Buffer conditions

Nine different conditions of pH and ionic strength were studied. For acidic conditions, 2-(*N*-Morpholino)ethanesulfonic acid or MES (Sigma-Aldrich) at pH 6.0 was used. For neutral conditions, Tris–HCl at pH 7.5 was used. For alkaline conditions, *N,N*-Bis(2-hydroxyethyl)glycine or Bicine (Sigma-Aldrich) at pH 8.3 was used. For ionic strength variation, the concentration of sodium chloride (NaCl) was varied from 0 M, 0.1 M, and 0.5 M. The buffer details are listed in Table 1.

**Table 1:** Compositions of buffers used in this study.

pH	Ionic strength (M)	MES (M)	Tris–HCl (M)	Bicine (M)	NaCl (M)	MgSO_4_ (M)	Gelatin (% w/v)
6.0	0.04	0.05	0	0	0	0.01	0.01
	0.14	0.05	0	0	0.1	0.01	0.01
	0.54	0.05	0	0	0.5	0.01	0.01
7.5	0.04	0	0.05	0	0	0.01	0.01
	0.14	0	0.05	0	0.1	0.01	0.01
	0.54	0	0.05	0	0.5	0.01	0.01
8.3	0.04	0	0	0.05	0	0.01	0.01
	0.14	0	0	0.05	0.1	0.01	0.01
	0.54	0	0	0.05	0.5	0.01	0.01

### Sample preparation for atomic force microscope

A mica substrate (V1 grade, Ted Pella) was cleaved and incubated in a 1% (3-Aminopropyl) triethoxysilane (APTS) (Sigma-Aldrich) solution in deionized water with 50 rpm shaking for 15 min. The mica was rinsed with deionized water and dried with nitrogen gas. A 50 μL droplet of the C22 phage solution with the titer of 10^12^ to 10^13^ pfu/mL was incubated with the APTS-treated mica substrate for 30 min prior to the AFM experiment.

### Atomic force microscopy imaging and nano-indentation

AFM imaging measurements were performed with a NanoWizard 3 BioScience AFM (JPK, Bruker). The images were collected in the non-contact mode (AC mode) under liquid condition. Data collected of the phage sample in liquid condition included shape, morphology, and dimension of the phage structure^17^. In the non-contact mode, the AFM cantilever with a nanometer-size tip at the end oscillates as the cantilever scans on the sample surface (the X and Y axes). The responsive oscillation amplitude was converted to the height of the sample (the Z axis).

In addition to the imaging capability in solution, the nano-mechanical properties of the virus particles can be assessed from their stiffness with the application of AFM-based nano-indentation technique performed under the force spectroscopy mode. A BioLever mini BL-AC40TS-C2 (Olympus) cantilever was used with the spring constant of 0.02 to 0.14 N/m determined by thermal tuning. The center region of the C22 phage particle was precisely located by choosing the center of the particle after the particle image was completely collected. A force of about 1 nN was applied in the center region of a single podovirus particle (Fig. 1a, left). The force-distance (FD) curve (Fig. 1a, right) on the C22 phage particle (blue curve) was collected along with the FD curve on the mica surface (red curve). The blue curve accounted for the indentation of both the particle and the cantilever while the red curve accounted for the indentation of the cantilever. Therefore, the distance difference between two curves indicated the indentation on the C22 phage particle. The slope calculated from the linear region of the FD curve was taken as the stiffness or spring constant value (force per distance in N/m). The stiffness extracted from indenting on the particle and the stiffness extracted from indenting on the mica surface were calculated based on the stiffness of the C22 phage particle described elsewhere^28^.

### AFM data analysis

For each buffer condition, 35–50 stiffness values were measured from 10–15 phage particles. From the empirical distributions of stiffness values, Gaussian fitting of distributions was performed using OriginLab software, which returns the center and the standard deviation of the distribution^15,28^. The center represents the stiffness of the C22 phage in a particular condition, and the standard deviation (s.d.) was used to calculate the standard error (s.e.m.) of the stiffness. The AFM results were collected within the first two weeks after the phage purification.

### Plaque assay protocol

An overnight culture of *R. solanacearum* strain RS3/1-1 at 28 °C and 230 rpm shaking was diluted to OD_600_ of 0.25 with fresh CPG medium. A 10 μL phage sample was added to 250 μL of diluted bacterial culture. After 45 min incubation, the sample was mixed with molten 0.45% agar in CPG medium and overlaid on a CPG plate containing 1.5% agar (Fig. 1b, left). After an overnight incubation at 28 °C, the number of plaques was recorded. An example of a plate with plaques indicating the lysis of the bacterial cells is shown in Fig. 1b, right.

The C22 phage titer was recorded for phage sampled at 14 day intervals of storage for a total of 182 days at 5–10 °C in the nine buffer conditions (Table 1). The first plaque assay was performed after incubating the C22 phage in the buffers for 24 h, and the titer from the first plaque assay for each buffer condition was used to normalize subsequent plaque assay measurements. The plaque assay was done in triplicate, and the average was calculated and normalized to the number of plaques after 1 day of storage, which was set to 100%.

### Adsorption test

This test quantified the proportion of C22 phage in each storage buffer bound to host cells. A modified plaque assay protocol was employed in which an overnight culture of *R. solanacearum* strain RS3/1-1 at 28 °C and 230 rpm shaking was diluted to OD_600_ of 0.25 with fresh CPG medium. A 10 μL phage sample in each storage buffer (Table 1) with known titer was added to 250 μL of diluted bacterial culture. After 45 min incubation, the sample tubes were centrifuged at 18,000×*g* for 10 min at 4 °C (Microfuge 2R, Beckman Coulter). 10 μL of the supernatant containing the C22 phage particles that were not attached to the host cells was added to 250 μL of diluted bacterial culture. After 45 min incubation, the sample was mixed with molten 0.45% agar in CPG medium and overlaid on a CPG plate containing 1.5% agar. After an overnight incubation at 28 °C, the number of plaques was recorded.

The number of the C22 phage particles initially added to the host cell culture and the number of the unattached C22 phage particles were calculated based on the phage titer and the added volumes. The assay was done in triplicate, and the average of the number of the C22 phage particles initially added to the host cell culture was calculated and normalized to 100%. The average number of the unattached C22 phage particles was calculated and normalized accordingly.

### Generalized additive models (GAM)

Relationship analysis using GAM was performed by function LinearGAM in package pyGAM, Python version 3.7.6^61^. LinearGAM returns Pseudo R-Squared which indicates the level of variation in dependent variable that can be explained by the independent variables along with the significance testing level (p-value) of using each independent variable. GAM has been widely applied to analyse the relationship of biological data in many fields^62–64^.

## Supplementary information

Supplementary information.
