# Supplementary material for: C22 podovirus infectivity is associated with intermediate stiffness
Source: Sci Rep. 2020 Jul 28;10:12604. doi: 10.1038/s41598-020-69409-w (PMC7387534; doi:10.1038/s41598-020-69409-w)
Supplement: Supplementary file 1 — Supplementary information. [file 41598_2020_69409_MOESM1_ESM.docx]

**Manuscript**

C22 podovirus infectivity is associated with intermediate stiffness

**Authors**

Udom Sae-Ueng^1,^*, Anjana Bhunchoth^1^, Namthip Phironrit^1^,

Alongkot Treetong^2^, Chaweewan Sapcharoenkun^2^,

Orawan Chatchawankanpanich^1^, Ubolsree Leartsakulpanich^1^,

and Penchit Chitnumsub^1^

^1^National Center for Genetic Engineering and Biotechnology,

National Science and Technology Development Agency, Pathum Thani, 12120, Thailand

^2^National Nanotechnology Center,

National Science and Technology Development Agency, Pathum Thani, 12120, Thailand

*udom.sae@biotec.or.th


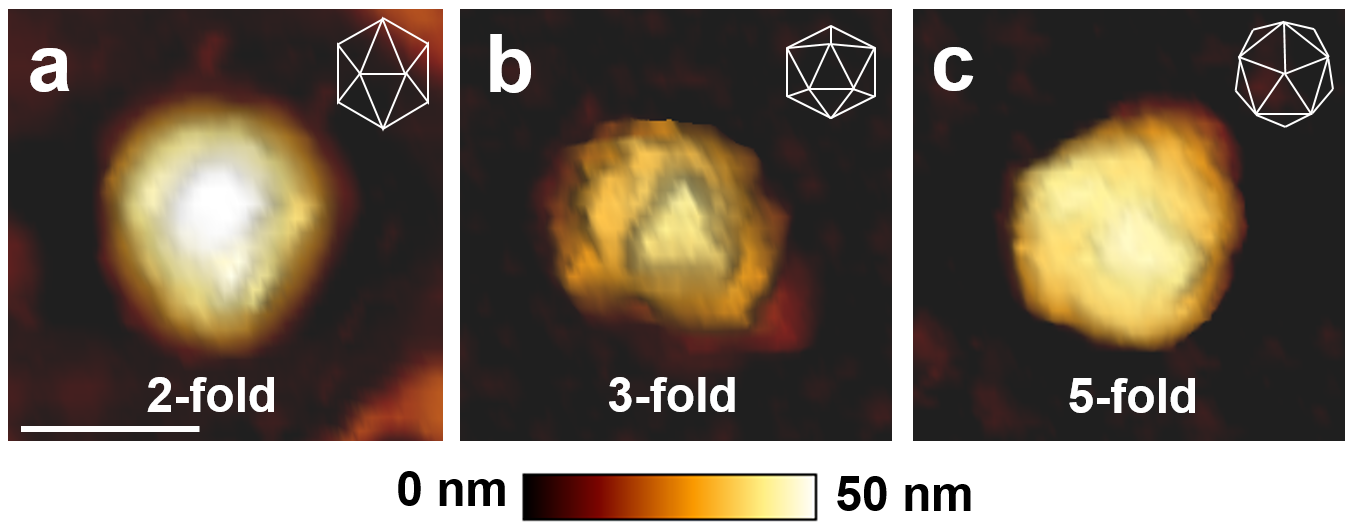


**Figure S1.** AFM images of the C22 phage particles with 2-fold, 3-fold, and 5-fold symmetry faces; white scale bar = 100 nm


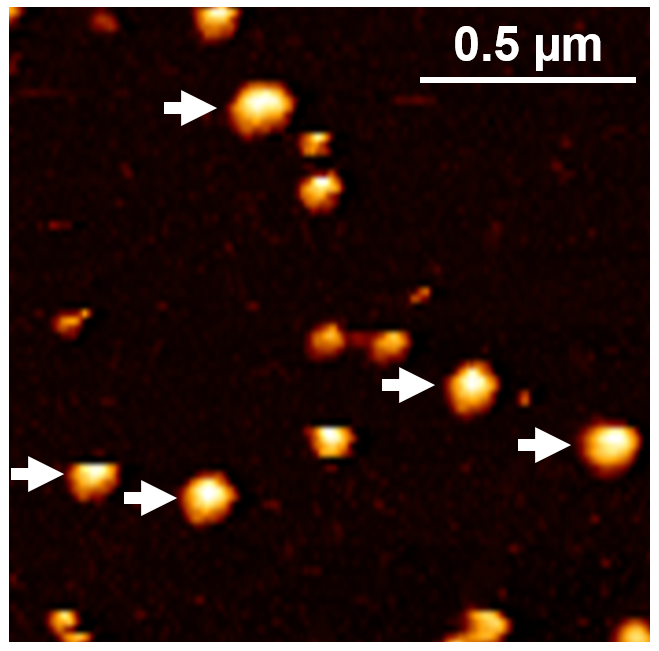


**Figure S2.** AFM image of the C22 phage in the buffer with pH 7.5 and ionic strength of 0.14 M showed no phage aggregation. No aggregation was observed by AFM in all nine buffers. White arrows indicate individual C22 phage particles.


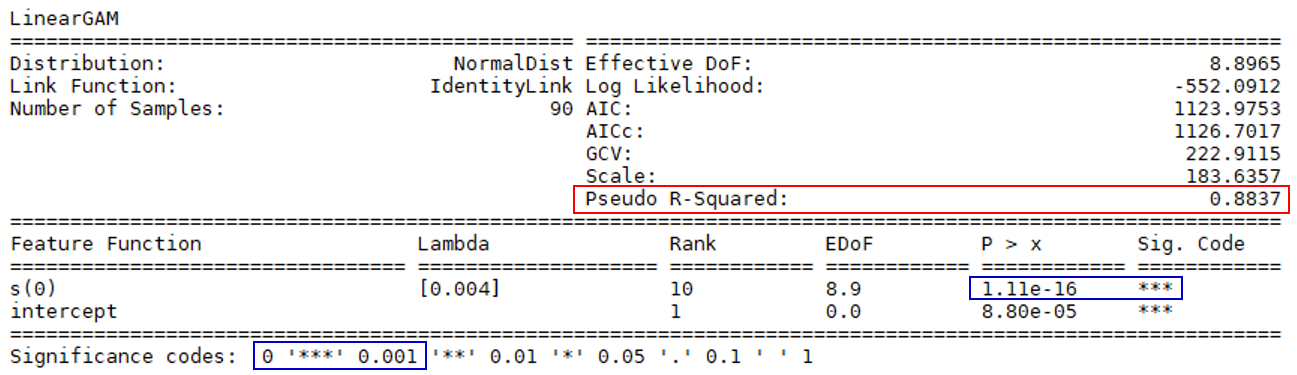


**Figure S3.** Relationship analysis between the stiffness and the infectivity data sets using GAM showed the Pseudo R-Squared (red rectangle) to be 0.8837, indicating strong relationship in the data sets. The values of R-Squared range from 0 to 1. The closer value to 1 indicates the stronger relationship that the variation in infectivity can be explained by stiffness. The p-values of each variable show the significance testing of having effects on the dependent variable. The p-value of 0.01 determines that such variable has a significant level of 99.99% in explaining the dependent variable. In this case, the p-value was between 0 and 0.001 (blue rectangles), suggesting that the changes in stiffness are greatly associated with the changes in infectivity.


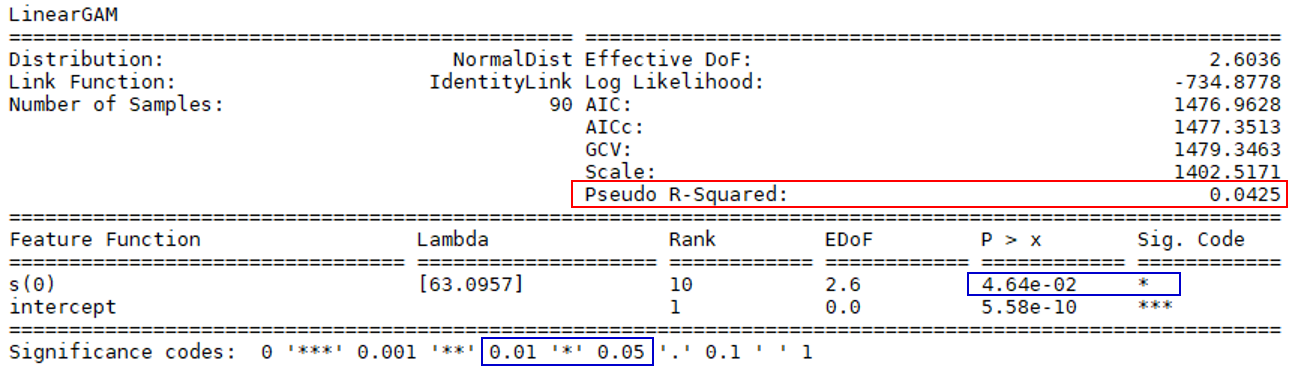


**Figure S4.** Relationship analysis between the stiffness and the adsorption data sets using GAM showed the Pseudo R-Squared (red rectangle) to be 0. 0425, indicating very weak relationship in the data sets. The values of R-Squared range from 0 to 1. The closer value to 1 indicates the stronger relationship that the variation in infectivity can be explained by adsorption. The p-values of each variable show the significance testing of having effects on the dependent variable. The p-value of 0.01 determines that such variable has a significant level of 99.99% in explaining the dependent variable. In this case, the p-value was between 0.01 and 0.05 (blue rectangles), suggesting that the changes in adsorption are associated with the changes in infectivity. However, their relationship is weak regarding to the small R-Squared.


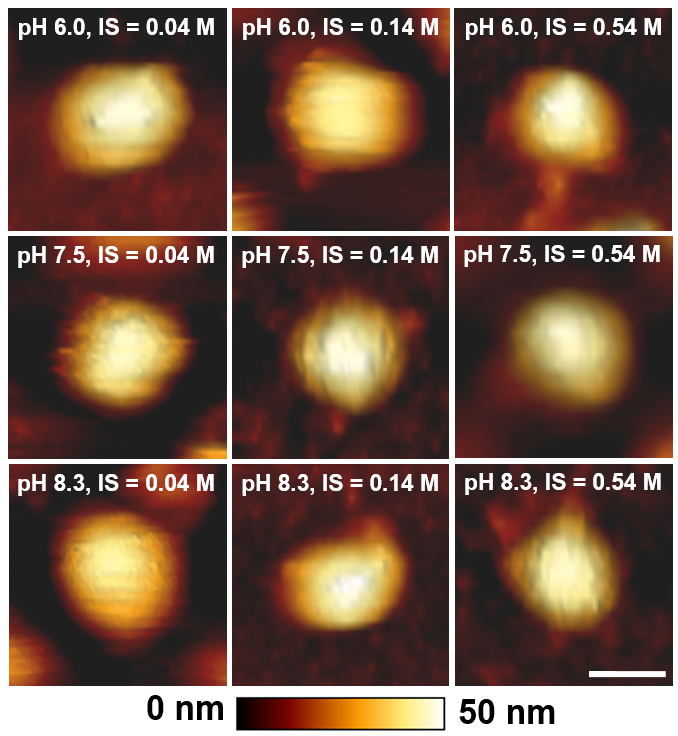


**Figure S5.** AFM images of the representative C22 phage capsids in each buffer condition show no apparent structural difference such as disassembly or deformation (white scale bar = 100 nm).


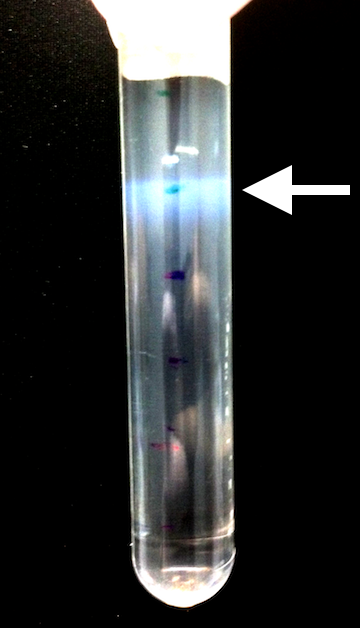


**Figure S6.** The band of the podovirus (white arrow) in an ultracentrifuge sucrose gradient (8.5 cm in length)

| pH | Ionic strength (M) | Stiffness (N/m) ± Standard error (s.e.m.) |
| --- | --- | --- |
| 6.0 | 0.04 | 0.10 ± 0.01 |
|  | 0.14 | 0.08 ± 0.01 |
|  | 0.54 | 0.11 ± 0.01 |
| 7.5 | 0.04 | 0.16 ± 0.01 |
|  | 0.14 | 0.13 ± 0.01 |
|  | 0.54 | 0.08 ± 0.01 |
| 8.3 | 0.04 | 0.05 ± 0.01 |
|  | 0.14 | 0.05 ± 0.01 |
|  | 0.54 | 0.04 ± 0.01 |

**Supplementary Table 1.** AFM data of C22 phage stiffness (N/m) in each buffer condition tested.

| pH | Ionic strength (M) | Orientations | Average *k* (N/m) ± Standard error (s.e.m.) |
| --- | --- | --- | --- |
| 6 | 0.14 | 2-fold | 0.09 ± 0.02 |
|  |  | 3-fold | 0.08 ± 0.01 |
|  |  | 5-fold | 0.07 ± 0.01 |
| 7.5 | 0.14 | 2-fold | 0.12 ± 0.01 |
|  |  | 3-fold | 0.13 ± 0.01 |
|  |  | 5-fold | 0.13 ± 0.01 |
| 8.3 | 0.14 | 2-fold | 0.06 ± 0.02 |
|  |  | 3-fold | 0.05 ± 0.01 |
|  |  | 5-fold | 0.05 ± 0.01 |

**Supplementary Table 2.** Stiffness of the C22 phage with different orientations in three buffers representing the stiffness in soft, intermediate, and hard ranges.

| **Soil properties** | **Soil sample 1** | **Soil sample 2** | **Soil sample 3** |
| --- | --- | --- | --- |
| Sand (%) | 11.57 | 47.81 | 62.5 |
| Silt (%) | 41.2 | 36.2 | 25.0 |
| Clay (%) | 47.23 | 15.99 | 12.5 |
| Texture | Clay | Loam | Sandy loam |
| pH | 7.56 | 7.72 | 6.7 |
| Avail. P (mg/kg) | 49.05 | 101.79 | 20.24 |
| Avail. K (mg/kg) | 175.01 | 27.41 | 126.48 |
| Avail. Ca (mg/kg) | 2956.88 | 1327.61 | 522.78 |
| Avail. Mg (mg/kg) | 214.81 | 225.43 | 95.55 |

**Supplementary Table 3.** Soil composition. Soil samples were collected from areas in which C22 phage was present.

| pH | Ionic strength (M) | The number of the C22 phage particles (%) attached to host cells ± Standard deviation (s.d.) |
| --- | --- | --- |
| 6 | 0.04 | 90.92 ± 3.73 |
|  | 0.14 | 91.05 ± 6.19 |
|  | 0.54 | 91.37 ± 4.39 |
| 7.5 | 0.04 | 91.78 ± 3.88 |
|  | 0.14 | 91.39 ± 4.51 |
|  | 0.54 | 88.60 ± 1.92 |
| 8.3 | 0.04 | 91.27 ± 2.76 |
|  | 0.14 | 93.06 ± 6.54 |
|  | 0.54 | 91.23 ± 2.19 |

**Supplementary Table 4.** Adsorption test data. The number of the C22 phage particles (%) attached to host cells in buffers with varied pH and ionic strength was calculated from the number of the C22 phage particles that was not attached to the host cells determined by the adsorption test assay.

| pH | Ionic strength (M) | Diameter of the C22 phage particles (nm) ± Standard deviation (s.d.) |
| --- | --- | --- |
| 6 | 0.04 | 39.8 ± 1.2 |
|  | 0.14 | 40.1 ± 1.1 |
|  | 0.54 | 40.2 ± 0.9 |
| 7.5 | 0.04 | 39.4 ± 0.9 |
|  | 0.14 | 40.5 ± 1.0 |
|  | 0.54 | 40.5 ± 1.3 |
| 8.3 | 0.04 | 40.3 ± 1.3 |
|  | 0.14 | 40.1 ± 1.2 |
|  | 0.54 | 40.7 ± 1.2 |

**Supplementary Table 5.** Diameter of C22 phage capsids in each buffer conditions tested.

| pH | IS (M) | C22 phage titer (%) | | | | | | | | | | | | | |
| --- | --- | --- | --- | --- | --- | --- | --- | --- | --- | --- | --- | --- | --- | --- | --- |
|  |  | 1D | 14 D | 28 D | 42 D | 56 D | 70 D | 84 D | 98 D | 112 D | 126 D | 140 D | 154 D | 168 D | 182 D |
| 6.0 | 0.04 | 100 | 125.0 | 88.0 | 97.2 | 92.6 | 100.0 | 90.7 | 80.6 | 87.0 | 99.1 | 94.4 | 75.9 | 78.7 | 86.1 |
|  | 0.14 | 100 | 105.4 | 85.5 | 86.3 | 101.2 | 85.5 | 84.6 | 75.3 | 77.2 | 73.4 | 71.0 | 69.4 | 72.7 | 76.3 |
|  | 0.54 | 100 | 87.9 | 103.5 | 74.2 | 93.1 | 84.5 | 65.5 | 77.6 | 74.2 | 84.5 | 79.3 | 74.2 | 86.2 | 85.0 |
| 7.5 | 0.04 | 100 | 114.5 | 69.3 | 59.7 | 66.1 | 59.0 | 63.9 | 50.0 | 48.4 | 36.0 | 25.3 | 15.8 | 11.9 | 7.9 |
|  | 0.14 | 100 | 78.5 | 61.3 | 59.1 | 50.5 | 51.4 | 56.7 | 71.0 | 48.4 | 52.2 | 11.0 | 9.2 | 5.5 | 3.4 |
|  | 0.54 | 100 | 94.7 | 95.8 | 80.7 | 88.1 | 96.3 | 96.1 | 81.9 | 79.8 | 86.6 | 77.2 | 75.1 | 76.2 | 76.6 |
| 8.3 | 0.04 | 100 | 67.2 | 61.4 | 60.0 | 51.4 | 41.4 | 37.1 | 18.6 | 22.9 | 20.0 | 12.9 | 16.9 | 11.4 | 9.6 |
|  | 0.14 | 100 | 80.0 | 57.0 | 51.4 | 44.0 | 40.1 | 43.4 | 15.6 | 22.8 | 9.7 | 12.9 | 8.7 | 4.8 | 5.2 |
|  | 0.54 | 100 | 70.5 | 37.1 | 46.2 | 34.8 | 31.1 | 22.0 | 6.8 | 3.0 | 2.6 | 1.3 | 1.0 | 1.3 | 1.3 |

**Supplementary Table 6.** Plaque assay data. The C22 phage titer (%) in buffers with varied pH and ionic strength (IS) was monitored. Phage titer was measured after one day of storage (1D) until day 182 (182D).
